# SPAG17 Is Required for Male Germ Cell Differentiation and Fertility

**DOI:** 10.3390/ijms19041252

**Published:** 2018-04-21

**Authors:** Elizabeth Kazarian, HyunYoung Son, Paulene Sapao, Wei Li, Zhibing Zhang, Jerome F. Strauss, Maria E. Teves

**Affiliations:** 1 Department of Obstetrics and Gynecology, Virginia Commonwealth University, Richmond, VA 23298, USA; ekazarian@mymail.vcu.edu (E.K.); sonh2@vcu.edu (H.S.); sapaopa@mymail.vcu.edu (P.S.); wei.li@vcuhealth.org (W.L.); zhibing.zhang@vcuhealth.org or gn6075@wayne.edu (Z.Z.); jerome.strauss@vcuhealth.org (J.F.S.); 2 Department of Biochemistry & Molecular Biology, Virginia Commonwealth University, Richmond, VA 23298, USA

**Keywords:** manchette, central pair complex protein, spermatogenesis

## Abstract

*Spag17* encodes a protein present in the axoneme central pair complex of motile cilia and flagella. A mutation in this gene has been reported to be associated with infertility caused by defects in sperm motility. Here, we report that *Spag17* knockout mice are infertile because of a severe defect in spermatogenesis. The histological evaluation of testis sections from mutant mice revealed seminiferous tubules with spermatogenesis arrested at the spermatid stage and cell debris in the cauda epididymis. The few sperm collected from the cauda epididymis were immotile and displayed abnormal tail and head morphology. Immunofluorescence analysis of *Spag17* knockout germ cells showed spermatids with abnormally long manchette structures and morphological defects in the head. Electron microscopy showed altered manchette microtubules, reduced chromatin condensation, irregular nuclear shape, and detached acrosomes. Additionally, the transport of proteins (Pcdp1 and IFT20) along the manchette microtubules was disrupted in the knockout elongating spermatids. Our results show for the first time that *Spag17* is essential for normal manchette structure, protein transport, and formation of the sperm head and flagellum, in addition to its role in sperm motility.

## 1. Introduction

*Spag17* encodes a protein present in the axoneme central pair complex (CPC) of motile cilia and flagella. *Spag17* is the orthologue of *PF6*, which was first identified and characterized in *Chlamydomonas reinhardtii* [1,2]. A large protein, PF6 is thought to be a scaffold for the assembly of the smaller components of the CPC C1a projection [3,4]. Domains near the C-terminus of PF6 are essential for flagellar motility and assembly of the C1a projection. The N-terminal half of the protein is not required for the assembly but it is important for stability of the C1a complex [2]. Although the murine *Spag17* gene also encodes a large protein (250 kDa) present in the CPC, the mammalian gene shows greater complexity in expression patterns and functions [5]. *Spag17* knockout mice develop a primary ciliary dyskinesia phenotype characterized by disrupted cilia motility in the trachea and brain. The animals show profound respiratory distress associated with lung fluid accumulation, hydrocephalus, and neonatal demise within 12 h of birth [6]. Additionally, the animals exhibit skeletal malformations associated with the functions that this gene plays outside of motile cilia [7]. Recently, twins from a consanguineous marriage with severe asthenozoospermia were found to have a predicted damaging homozygous missense mutation (R1448Q) affecting the C-terminus of the SPAG17 protein [8]. This finding supports the notion that the C-terminus region plays a key role in flagellar motility, as predicted from studies in model organisms.

Spermatogenesis constitutes a well-regulated process for male germ cell differentiation. It can be divided into three phases: proliferation of spermatogonia by mitosis, meiotic division of spermatocytes, and the final differentiation phase called spermiogenesis [9]. During spermiogenesis, haploid round spermatids undergo substantial changes, including chromatin condensation, elongation of the sperm head, and building of the flagella. The manchette, a transient organelle consisting of microtubules, actin filaments, and associated motor proteins, plays a central role during spermiogenesis [10,11]. It has been proposed that the manchette contributes to the transport of proteins by intramanchette transport (IMT), a mechanism that resembles intraflagellar transport (IFT) in the flagellum [11]. In this study, we evaluated the functions of the *Spag17* gene in male germ cells. Our findings reveal for the first time that *Spag17* is essential for male germ cell differentiation in addition to sperm motility.

## 2. Results

### 2.1. Localization of SPAG17 in Male Germ Cells

*Spag17* mRNA expression during the first wave of spermatogenesis has been detected on day 16, when pachytene spermatocytes are present, and coincides with the initiation of transcription during the meiosis phase [12,13]. Immunohistochemical studies on testis sections have shown that SPAG17 expression is more intense in the cytoplasm of round and condensing spermatids [13].

**Figure 1:**
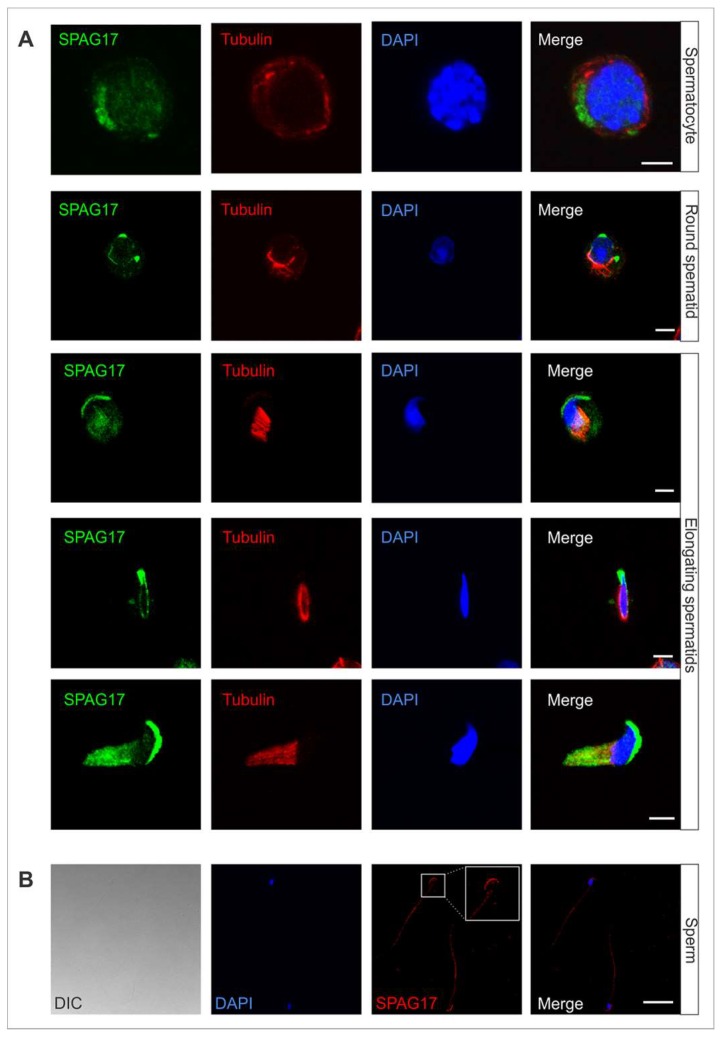
Distribution of SPAG17 in germ cells. Immunofluorescence was performed on (**A**) mixed germ cells collected from wild-type mice. Acetylated α-tubulin was used as a marker for microtubules. The images show SPAG17 in the cytoplasm of spermatocytes and round spermatids and co-localization with manchette microtubules in elongating spermatids. Scale bars = 5 µm. (**B**) In sperm, SPAG17 decorates the acrosome and the tail. DIC, Differential interference contrast. Scale bar = 25 µm.

**Figure 2:**
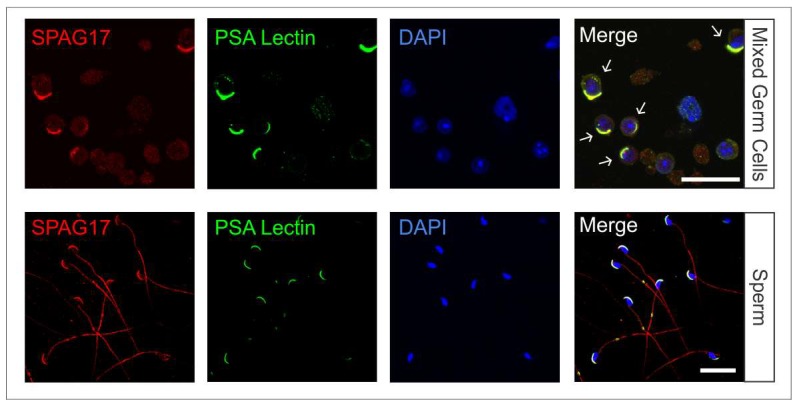
SPAG17 co-localizes with Golgi and acrosome structures. Immunofluorescence was performed on germ cells and sperm collected from wild-type mice. *Pisum sativum agglutinin* (PSA) lectin was used as a marker for Golgi and acrosome. The images show SPAG17 co-localization with Golgi-derived structures during spermiogenesis. The arrows indicate germ cells with co-localization of SPAG17 and PSA. Scale bars = 25 µm.

Mixed germ cells from wild-type 6-week-old mice were isolated and co-immunostained with anti-SPAG17 antibody (green) and anti-acetylated tubulin antibody (red) as a microtubule marker (Figure 1A). SPAG17 was present in the cytoplasm of germ cells and co-localized with the manchette microtubule structures throughout steps 8 to 14 in elongating spermatids. In sperm, SPAG17 decorated the tail and acrosome (Figure 1B). To confirm SPAG17 co-localization with acrosome and Golgi, a *Pisum sativum agglutinin* (PSA) lectin was used as a marker for these structures. Figure 2 shows the co-localization of SPAG17 with Golgi vesicles and acrosome structures. The specificity of this antibody was further confirmed using samples from *Spag17* knockout mice (Supplementary Figures S1).

### 2.2. Deletion of Spag17 Causes Male Infertility

**Figure 3:**
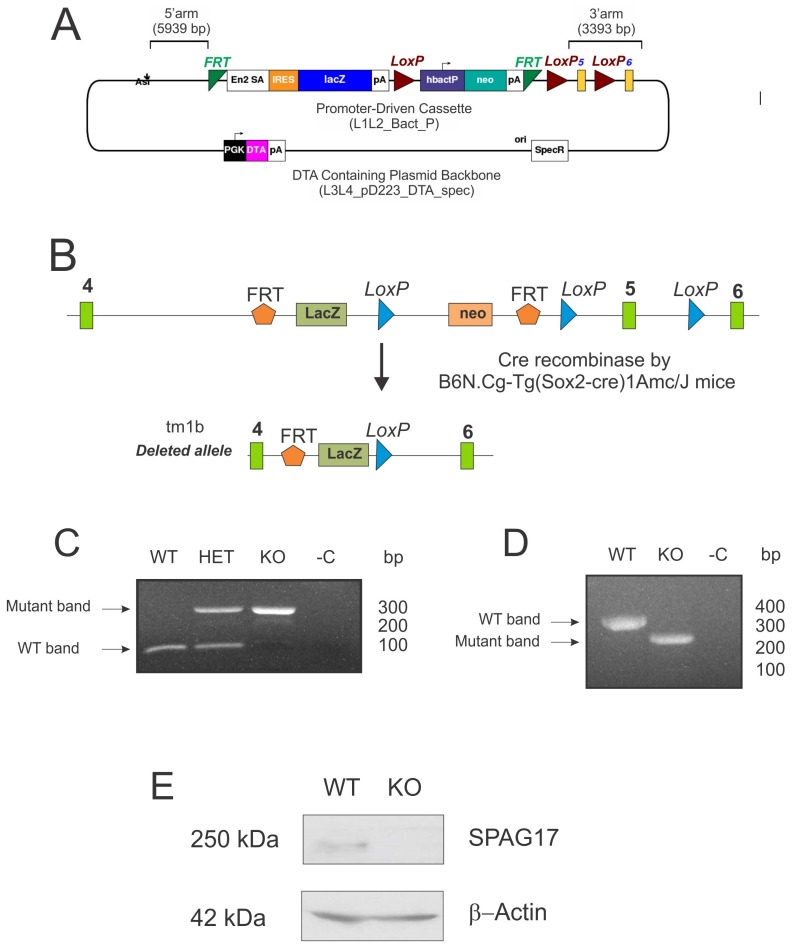
Targeted disruption of the *Spag17* gene in the testis. (**A**) Schematic representation of the L1L2_Bact_P cassette inserted in Chromosome 3. (**B**) Schematic representation of the Cre-recombination for the deletion of exon 5. (**C**) Genotyping results. (**D**) mRNA expression of *Spag17* was evaluated by RT-PCR in extracts of testis from wild-type and knockout mice. (**E**) Representative western blot showing the absence of SPAG17 protein in the testis from knockout mouse. WT, wild-type; HET, heterozygous; KO, homozygous knockout.

To investigate the function of *Spag17*, we first developed a global deletion of *Spag17* using a human cytomegalovirus promoter (CMV)-Cre mouse [6]. The homozygous knockout mice died within 12 h of birth [6] making it impossible to perform fertility studies. Therefore, we used another conditional deletion approach. We studied B6N(Cg)-*Spag17 ^tm1b(KOMP)Wts1^*/J mice developed by Jackson Laboratories which have a disruption of exon 5 of the *Spag17* gene driven by the *Sox2* promoter. *Sox2* is known to be expressed throughout epiblast cells (E6.5). In the testis, *Sox2* is expressed around E10 in primordial germ cells [14]. Figure 3A,B show the vector and the strategy for the deletion of exon 5. The mice were genotyped by PCR using a combination of specific primers. The top band in the representative gel belongs to the mutant allele, and the lower band to the wild-type allele (Figure 3C). *Spag17* gene expression in the testis of mutant and wild-type mice was evaluated by reverse transcription-polymerase chain reaction (RT-PCR) (Figure 3D), as previously described [6]. DNA sequencing revealed the absence of exon 5 and the presence of a premature stop codon after the deletion (Supplementary Figure S2). Protein expression was evaluated by western blotting, as shown in Figure 3E, documenting the absence of SPAG17 in homozygous knockout mice.

It should be noted that this line of *Spag17* knockout mice differs from the line we originally created [6] because it was generated using *Sox2*-Cre as opposed to CMV-Cre. The *Sox2* and CMV promoters drive different Cre expression patterns, resulting in differential disruption of *Spag17* during embryonic development, accounting for the variation in phenotypes. The CMV-Cre mice create a more global disruption of the *Spag17* gene that prevents survival beyond the immediate neonatal period, precluding the study of spermatogenesis [6]. In addition, the backgrounds of the mice studied in this report (C57BL/6NJ) differ from that of the *Spag17* knockout model we created (mixed C57BL/6J-129S4/SvJ). This report focuses on the characterization of the male infertility phenotype in the *Spag17* knockout mice.

Mating experiments were performed to test fertility. Adult wild-type and knockout males were bred to wild-type females. Both wild-type and knockout males were sexually active and produced vaginal plugs. After 2 months of continuous mating, none of the females that mated with *Spag17* knockout males became pregnant or gave birth to any offspring (Table 1). The *Spag17* knockout females that mated with wild-type males became pregnant and gave birth during their first pregnancy. However, by the second pregnancy the females died at term, evidently from obstructed labor. The weight of the testis of *Spag17* knockout and wild-type mice were similar (WT = 132.9 ± 7.0 mg; KO = 121.9 ± 6.8 mg, *n* = 5, *p* > 0.05), and male accessory organs were not grossly different in appearance, indicating that the deletion of *Spag17* did not likely affect gonadotropin production or testicular steroidogenesis.

**Figure 4:**
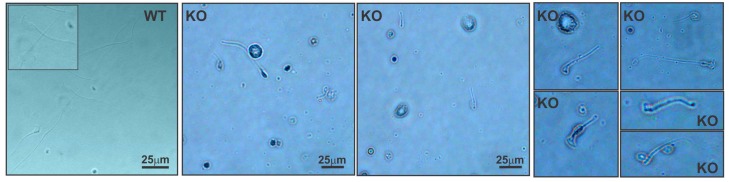
Disruption of the *Spag17* gene causes male infertility. Cauda epididymal sperm from *Spag17* knockout mice have deformities in the head and tail. WT, wild-type; KO, knockout.

**Figure 5:**
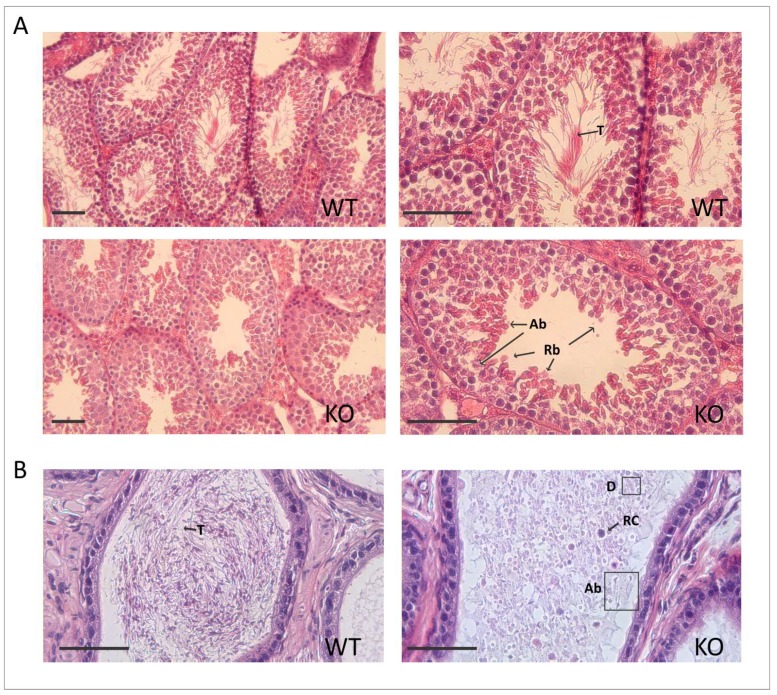
The *Spag17*-deficient mice have severe defects in spermatogenesis. (**A**) Testis sections from knockout mice show major defects at spermatids stages 11–12. Abnormal head shapes of elongating spermatids are observed. Sperm tails are absent in the tubular lumen. (**B**) Cauda epididymis shows degenerated cell debris in the knockout epididymis. Indicated with black arrows and boxes: T, tail; Ab, abnormal; Rb, residual bodies; RC, round cells; D, debris. Scale bars = 100 µm.

To determine whether the failure of male fertility was associated with poor sperm quality, sperm from the cauda epididymis was collected. The knockout mice had reduced sperm counts (Table 1), and the few sperm recovered after washing the cauda epididymis displayed morphological abnormalities. Spermatozoa from the knockout mice had short flagella and defects in the sperm heads (lack of head or irregular head shapes and absence of the typical hook-shaped appearance, Figure 4), suggesting that sperm production was affected. In addition, the knockout spermatozoa showed no motility (Supplementary Video S1). Histological analysis of testis sections revealed disrupted spermatogenesis at the spermatid stage. The most notable defects where found at stages XI–XII, where elongating spermatids in steps 11–12 showed an abnormally elongated head shape. Additionally, sperm tails were absent in the tubular lumen at stages VII–VIII (Figure 5A). The evaluation of the cauda epididymis showed mature spermatozoa with long tails in the lumens of the adult wild-type epididymis, whereas, in the knockout mice, normal sperm were absent (Figure 5B). Indeed, degenerated cell debris was observed in the knockout epididymis.

### 2.3. Spag17 Knockout Mice Display Defects in Manchette Structure and in the Morphology of the Sperm Head, Acrosome, and Tail

**Figure 6:**
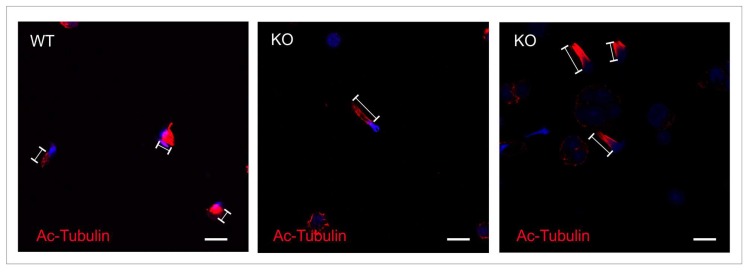
The manchette is abnormally elongated in *Spag17* knockout mice. Germ cells were stained with anti-acetylated α-tubulin antibody to visualize manchette microtubules. The distance from the perinuclear ring to the caudal side of the head is longer in the knockout mice. WT, wild-type; KO, knockout. Scale bar = 10 µm.

**Figure 7:**
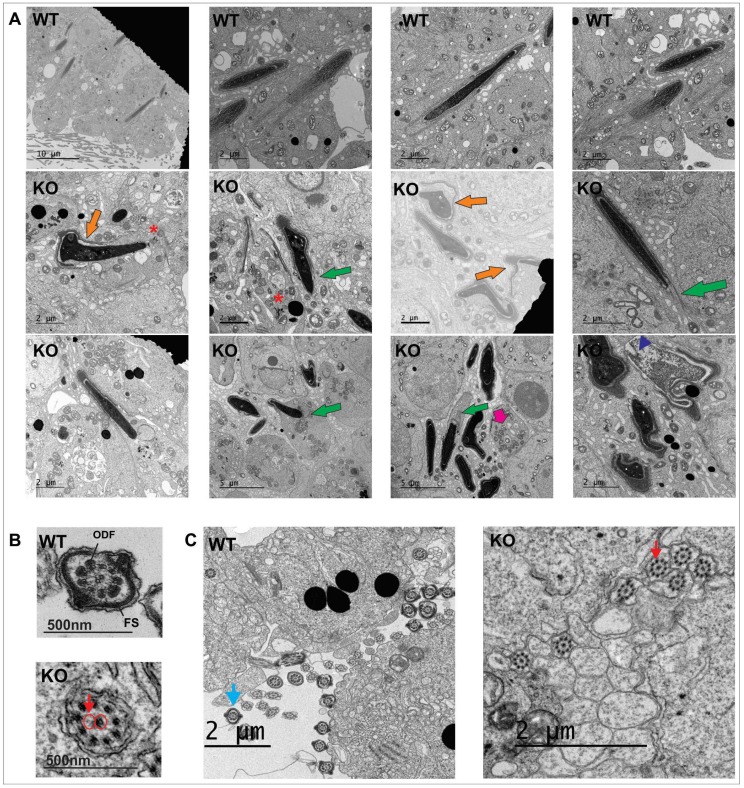
Structural analysis of elongating spermatids by TEM. (**A**) Wild-type elongating spermatids show normal assembly of the manchette and chromatin condensation. The morphology of the head and acrosome develops normally as well. Elongating spermatids from knockout mice have several defects. The manchette structure is disorganized (green arrows) or absent. Chromatin condensation is affected (blue arrowhead) as well as the nucleus and acrosome shapes. The orange arrows show detached acrosomes; * shows dispersed outer dense fiber material. The pink arrows show disorganized mitochondria. (**B**) TEM microscopy from testis samples shows some axonemes missing one CP microtubule (9 + 1) indicated by the red arrow; however, the majority of the axonemes show the 9 + 2 arrangement of their microtubules. (**C**) Fibrous sheath and outer dense fibers were observed in the wild-type testis (the blue arrow points to a representative flagellum showing these structures). However, these structures were not observed around the axoneme in the knockout flagella. Red arrow indicate 9 + 1 axoneme. WT, wild-type; KO, knockout; ODF, outer dense fibers; FS, fibrous sheath.

During spermiogenesis, haploid round spermatids undergo substantial changes, including chromatin condensation, elongation of the sperm head, and building the flagella. The manchette is a transient skirt-like structure that plays an important role in the elongation and chromatin condensation of the spermatid nucleus, as well as in the growth of the axoneme [15]. Spermatids were stained with anti-acetylated tubulin antibody to visualize the manchette microtubules. Figure 6 shows abnormal extension of the manchette in elongating spermatids from *Spag17* knockout mice compared to elongating spermatids from wild-type mice. These results suggest that the absence of SPAG17 affects the manchette microtubule structure. Transmission electron microscopy studies revealed wild-type elongating spermatids with normal assembly of the manchette and chromatin condensation. The morphology of the head and acrosome was normally as well. However, elongating spermatids from the knockout mice had several defects, including disorganized manchette structure (Figure 7, green arrows) or absence of the manchette in some cells and altered chromatin condensation (blue arrowhead) as well as nuclar shape. Additionally, there was a defect in the attachment of the acrosome in some cells (orange arrows). Since SPAG17 also localizes in the CPC [13], we assessed whether there was a disruption in the CPC microtubules. EM microscopy from testis samples showed some axonemes missing one CP microtubule (9 + 1), but the majority of the axonemes had a 9 + 2 arrangement of microtubules (Figure 7B,C). However, fibrous sheath and outer dense fibers were not observed (Figure 7B,C), consistent with the thin appearance of the sperm flagella (Figure 4).

### 2.4. Intramanchette Transport Is Disrupted in Spag17 Knockout Mice

**Figure 8:**
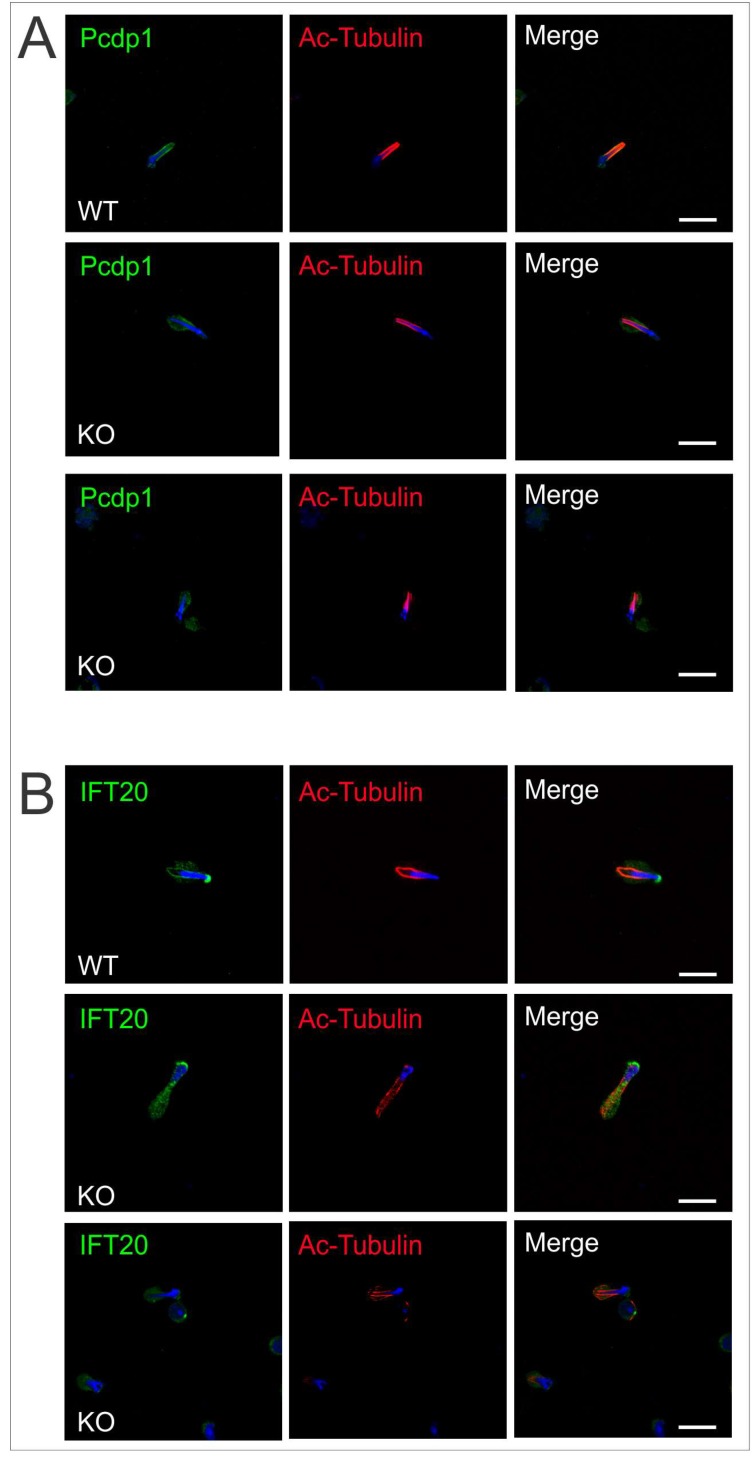
Pcdp1 and IFT20 localization in the manchette is dependent on SPAG17. Germ cells from wild-type and knockout mice were collected from adult mice and stained for proteins associated to manchette structures. (**A**) Pcdp1 and (**B**) IFT20 failed to localize to the manchette in the elongating spermatids from *Spag17* knockout mice, and their localization appears diffused in the cytoplasm. Scale bar = 10 µm.

The manchette is essential for the intracellular transport of proteins and vesicles along the microtubule scaffolding. These cargo travel from the Golgi apparatus to the acroplaxome covering the nucleus and to the developing axoneme. The delivery of proteins is achieved along the microtubules with the assistance of motor proteins (kinesin and dynein), which are linked to cargo proteins [15]. Results from previous mouse models have underlined the importance of this transport process during male germ cell differentiation and have identified several protein complexes that attach to manchette microtubules [11]. We previously showed that Pcdp1, a central pair protein that, like SPAG17, also localizes to the C1 microtubule [16], is affected in the absence of SPAG17 [6]. Immunofluorescence results showed that Pcdp1 protein is present in the cytoplasm of elongating spermatids. However, Pcdp1 localization along the manchette structure in the knockout elongating spermatids was altered compared to the wild-type controls (Figure 8A). This finding suggests that SPAG17 may recruit Pcdp1 to the manchette microtubule directly or indirectly.

Intraflagellar transport (IFT) involves multi-protein complexes. Proteins in these complexes have motifs like WD-40 repeats, TPRs (tetratrico peptide repeat), and coiled-coil domains important for protein–protein interaction. These proteins associate with motor proteins and move along the microtubules to carry precursors needed for ciliary/flagellar assembly. Some IFT proteins also localize to the manchette [17,18,19]. IFT20 is an important protein in IFT complex B. Conditional *Ift20* knockout mice develop male infertility due to defects in spermatogenesis and it has been proposed to be important for intramanchette transport by Zhang and colleagues [18]. Thus, we investigated whether there was any change in the localization of IFT20 in the *Spag17* knockout mice. Figure 8B shows that elongating spermatids from *Spag17* knockout mice expressed IFT20. However, the protein no longer localized to the manchette. In summary, these results suggest that SPAG17 is important for intramanchette transport.

## 3. Discussion

In this work, we used a conditional knockout mouse model to study the function of *Spag17* in male germ cells. The manchette and the axoneme are microtubular structures which contain α- and β-tubulin. During spermatogenesis, the manchette is assembled during step 8 spermatids, forming a platform between the perinuclear ring around the nucleus and the elongated sperm axoneme [17]. Disassembly of the manchette takes place around step 14, before the formation of the mid-piece in the flagellum [11]. In the mouse, the manchette structure moves toward the tail neck region in a zipper-like fashion. This constriction has been suggested to contribute to the elongation and shaping of the spermatid head [11]. A number of mutant mouse models affecting manchette formation have been generated [11]. Elongation of the manchette microtubules and defects in sperm head, acrosome, and tail morphology appear to be common phenotypes. Although there is a range in the severity of the phenotypes of the mutants, the defects shown by the *Spag17* knockout germ cells resemble the phenotypes shown by other mouse mutants affecting proteins associated with the manchette [11]. The elongating spermatids from *Spag17* knockout mice display defects in acrosome attachment, head, and tail, as well as defects in manchette structure. Moreover, SPAG17 disruption affects the recruitment of proteins to the manchette (Pcdp1 and IFT20), suggesting a role in the intramanchette transport of cargo proteins. It is not clear yet which are the direct interacting partners of SPAG17 and how this protein interacts with IFT transport. SPAG17 has been reported to directly interact with SPAG6 and SPAG16L (other CPC proteins) [13], but a direct interaction with IFT proteins has not yet been evaluated. Further studies of this protein could provide valuable information regarding spermatid-specific factors controlling fertility that might be used for contraceptive targeting.

*Spag17* is known to be a cilia/flagella-related gene. Previous studies in *Chlamydomonas* have shown that PF6, the orthologue of the mammalian SPAG17 protein, is essential for flagellar motility [2]. Twin males with severe asthenozoospermia were described with a homozygous missense mutation in the *SPAG17* gene [8]. This finding implies that a C-terminal domain in SPAG17 is critical for sperm motility. Other domains appear to have different roles in spermatogenesis, consistent with our assertion that the mammalian *SPAG17* gene is complex and has pleiotropic functions [5]. We have shown that *Spag17* plays a role beyond motile cilia and flagella and influences bone growth and mineralization [7]. Recently, a homozygous *SPAG17* missense mutation was found in a 7-year-old boy. The patient had multiple congenital anomalies including brain and bone deformities similar to those observed in the global *Spag17* knockout mouse we developed [20]. Although the patient had an additional mutation in a primary cilia-related gene (*WDR35*), the phenotype of this patient cannot be explained entirely by this mutation [21], providing evidence for the involvement of *SPAG17* in the biology of skeletal cells. The fertility status of this subject is not yet known. The mechanisms underlying the pleiotropic functions of *SPAG17* are not yet known and deserve further investigation.

In conclusion, we propose that the *Spag17* gene is important for male fertility and that it plays roles in spermatogenesis beyond regulating sperm motility. We showed that *Spag17* localizes to structures other than the central pair apparatus of the axoneme, decorating manchette microtubules, Golgi, and acrosome structures in male germ cells. Deletion of this gene in germ cells significantly impaired spermatogenesis, revealing new functions for this gene in male germ cells differentiation. 

## 4. Materials and Methods

### 4.1. Animals

The experiments were conducted in accordance with the SSR’s specific guidelines and standards. The animal protocol AM10297 was approved on 5/1/17 by the Virginia Commonwealth University Institutional Animal Care and Use Committee. Heterozygous B6N(Cg)-*Spag17 ^tm1b(KOMP)Wts1^*/J (Stock No. 026485) mice from Jackson Laboratories were used to generate homozygous mice with disrupted expression of the *Spag17* gene. The knockout/reporter mutant of the *Spag17* gene has been generated by the Knockout Mouse Phenotyping Program (KOMP2) at The Jackson Laboratory. The L1L2_Bact_P cassette was inserted on Chromosome 3. The cassette is composed of an FRT site followed by lacZ sequence and a loxP site. This first loxP site is followed by neomycin under the control of the human β-actin promoter, SV40 polyA, a second FRT site, and a second loxP site. A third loxP site is inserted downstream of the targeted exon 5. The critical exon is thus flanked by loxP sites. The mouse strain was generated by the Knockout Mouse Phenotyping Program (KOMP2) at The Jackson Laboratory using embryonic stem cells provided by the International Knockout Mouse Consortium. The construct was introduced into C57BL/6N-derived JM8.N4 embryonic stem (ES) cells, and correctly targeted ES cells were injected into B6(Cg)-Tyrc-2J/J (Stock No. 58) blastocysts. The resulting chimeric males were bred to C57BL/6NJ (Stock No. 005304) females and then to B6N.Cg-Tg(Sox2-cre)1Amc/J mice (Stock No. 014094) to remove the floxed neomycin cassette and critical exon sequences. The resulting offspring were bred to C57BL/6NJ mice to remove the Cre-expressing transgene.

### 4.2. Mixed Germ Cell Isolation

The testes from adult wild-type and *Spag17* knockout mice were de-capsulated and placed in 5 mL DMEM containing 0.5 mg/mL collagenase IV (Sigma-Aldrich, St. Louis, MO, USA) and 1.0 mg/mL DNase I (Sigma-Aldrich, St. Louis, MO, USA), then incubated for 30 min at 32 °C to dissociate the testicular cells, and then centrifuged for 5 min at 2000 rpm. Dispersed mixed testicular cells were fixed by 15 min incubation in 4% paraformaldehyde/PBS (containing 0.1 M sucrose) at room temperature and then washed three times with PBS. The cells were re-suspended in 10 mL PBS, spread on SuperFrost/Plus microscope slides (Fisher Scientific, Pittsburgh, PA, USA), and allowed to semi-dry.

### 4.3. Sperm Isolation

Sperm were collected after swimming out from the cauda epididymides into PBS, at 35 °C for 10 min. An aliquot from the sperm suspensions was observed under the microscope for motility and morphology evaluation.

### 4.4. Immunofluorescence

The cells were permeabilized with 1% Triton X-100 (Sigma-Aldrich, St. Louis, MO, USA) for 5 min 37 °C, washed with PBS three times, and blocked at room temperature for 1 h with 10% goat serum (Vector Laboratories, Inc., Burlingame, CA, USA) in PBS. Then, the cells were incubated overnight with rabbit anti-SPAG17 antibodies previously developed for our lab [13], and mouse anti-acetylated tubulin (Sigma-Aldrich, St. Louis, MO, USA) at 4 °C. (See Supplemental Table S1). After washing, the cells were incubated with anti-rabbit Alexa Fluor 488-labeled, anti-rabbit Cy3-labeled, or anti-mouse Alexa Fluor 594-labeled secondary antibodies (Jackson ImmunoResearch laboratory Inc., Grove, PA, USA). For acrosome staining, the cells were incubated 15 min with 20 µg/µL PNA lectin. The cells were subsequently washed with PBS, mounted with VectaMount with DAPI (Vector Laboratories, Inc., Burlingame, CA, USA), and sealed with nail polish. Images were captured by Zeiss LSM 700 confocal laser-scanning microscope.

### 4.5. Mouse Genotyping

The mice were genotyped by PCR using the primers: forward 5′-CTGTCTTGATGAGAATGTAATG-3′ (this sequence is present in the wild-type genomic DNA but absent in the mutant mouse DNA), reverse 5′-GAGTGAGCAACTTTCCTCAGGAG-3′ (this sequence is present in the wild-type genomic and mutant mouse DNA), and forward 5′-CCCTGAACCTGAAACATAAA3′ (this sequence is present upstream of the first LoxP site in the vector sequence). For the wild-type band, the expected PCR product is 96 bp. The mutant band will be larger because of an extra sequence present in the vector (PCR product around 300 bp after Cre-recombination).

### 4.6. RT-PCR

RNA was isolated from the mouse testes with TRIzol (Invitrogen, Carlsbad, CA, USA), total RNA was reversed transcribed with RETROscript kit (Ambion, Austin, TX, USA) according to the manufacturer’s instructions, and the cDNAs were used for PCR, using primer sets specific for mouse *Spag17*. Forward primer: 5′-CTTGAAGTGTCAACTTCTCC-3′ (located within exon 4); reverse primer: 5′-CCAAGCTCAGTCATAATGGCC-3′ (in exon 6). The amplification products were resolved on 1% agarose gel stained with ethidium bromide. The results presented are representative of three wild-type and knockout animals. DNA bands were cut, and DNA was extracted with Gel extraction kit (Quiagen, Hilden, Germany), following the manufacturer’s protocol. The amplified cDNA was submitted for sequencing to the VCU Nucleic Acids Research Facility. 

### 4.7. Histology

The testes and epididymides of adult mice were dissected and fixed in a 4% formaldehyde solution in PBS, paraffin-embedded, and sectioned into 5 μm slices. Hematoxylin and eosin staining was carried out using standard procedures. Histology was examined using a BX51 Olympus microscope (Olympus Corp., Melville, NY, USA; Center Valley, PA, USA), and photographs were taken with the ProgRes C14 camera (Jenoptik Laser, Germany).

### 4.8. Transmission Electron Microscopy (TEM)

The mouse testes were fixed in 4% paraformaldehyde, 2% glutaraldehyde in 0.1M cacodylate buffer, and post-fixed in 1% OsO_4_. Sections (80 nm), stained with lead citrate and uranyl acetate, were imaged with a JEOL JEM-1400 (Plus) transmission electron microscope equipped with a high-sensitivity sCMOS camera.

## Figures and Tables

**Table 1:** Sperm count and fertility.

Genotype	Cell Count (1 × 10 ^−6^/mL)	Male Fertility	Litter Size
WT	5.73 ± 0.79	6/6	8.5 ± 0.9
KO	0.01 ± 0.003 *	0/6	0

To test fertility, sexually mature WT and KO males were bred to WT females. * Significantly different vs. WT, *p* < 0.05.

## Supplementary Material

Supplementary materials are available online at http://www.mdpi.com/1422-0067/19/4/0/s1.

## Abbreviations

IMT	Intramanchette transport
IFT	Intraflagellar transport
CPC	Central pair complex
