# Supplementary material for: SPAG17 Is Required for Male Germ Cell Differentiation and Fertility
Source: Int J Mol Sci. 2018 Apr 21;19(4):1252. doi: 10.3390/ijms19041252 (PMC5979577; doi:10.3390/ijms19041252)
Supplement: Supplementary file 1 [file ijms-19-01252-s001.zip › ijms-287252 supplementary/ijms-287252 supplementary caption.docx]

Supplementary Figure 1: Specificity of SPAG17 antibody for immunofluorescence was evaluated using samples from *Spag17* knockout mice.

Supplementary Figure 2: Deletion of exon 5 results in a premature stop codon disrupting the expression of SPAG17 protein. A) Translated nucleotide sequence from testis samples from knockout mice. B) Representative nucleotide sequence result from testis samples from knockout mouse.

Supplementary Table 1: List of antibodies used for this study.

Supplementary video 1: Representative video showing a sperm sample collected from cauda epididymis from a *Spag17* knockout mouse.
