# Supplementary material for: SPAG17 Is Required for Male Germ Cell Differentiation and Fertility
Source: Int J Mol Sci. 2018 Apr 21;19(4):1252. doi: 10.3390/ijms19041252 (PMC5979577; doi:10.3390/ijms19041252)

A

```
atggcacccaagaaggagaaacctaccgggagcgcaaactataagatatgggaaccttcc
 M  A  P  K  K  E  K  P  T  G  S  A  N  Y  K  I  W  E  P  S
ctcatagctgcacacttgaaccagaatgattggaaggcctccatcgccttcgtggttggg
 L  I  A  A  H  L  N  Q  N  D  W  K  A  S  I  A  F  V  V  G
aaccgagttgaggatgatcttctcattcatgcccttgacctggctgttcgactccctcag
 N  R  V  E  D  D  L  L  I  H  A  L  D  L  A  V  R  L  P  Q
cggaaactcttcagtattgtgtcatgggaagacattctgcagcagatggatgaaatacag
 R  K  L  F  S  I  V  S  W  E  D  I  L  Q  Q  M  D  E  I  Q
tcacttgctgaaagtgcttcagctaaaaaggggaaaaagcccacatctgtcaatttaccg
 S  L  A  E  S  A  S  A  K  K  G  K  K  P  T  S  V  N  L  P
ttacattacgaggtgttcctggcagcaaagataatcatggagagtggagagaaactaacc
 L  H  Y  E  V  F  L  A  A  K  I  I  M  E  S  G  E  K  L  T
ttaccattgatagggaaactcttgaagtgtcaacttctccatattaaatccaaggaccag
 L  P  L  I  G  K  L  L  K  C  Q  L  L  H  I  K  S  K  D  Q
cagagacgagaaaatgaaaagaaggtcccaggtcccgaaaaccaaagaagaagaaccctа
 Q  R  R  E  N  E  K  K  V  P  G  P  E  N  Q  R  R  R  T  L
acaaagaggacaagcggcctcgcacagccttcactgctgagcagctccagaggctcaagg
 T  K  R  T  S  G  L  A  Q  P  S  L  L  S  S  S  R  G  S  R
ctgagtttcagaccaacagatgatgagccagacgatggtgcccaatattacattattgtt
 L  S  F  R  P  T  D  D  E  P  D  D  G  A  Q  Y  Y  I  I  V
gtgggcttcaacaatcctcagctattggccattatgactgagcttggaccctacttttc
 V  G  F  N  N  P  Q  L  L  A  I  M  T  E  L  G  P  Y  F  F
tgttttgctccccctacttagccccctgcatgaaaatggataaattgccgctttgatgat
 C  F  A  P  L  L  S  P  L  H  E  N  G STOP I  A  A  F  D  D
aactgatttatattgggattgaaccgtctgtgccaatggctcgttttagacccgtaatgc
 N STOP F  I  L  G  L  N  R  L  C  Q  W  L  V  L  D  P STOP C
ttgtcatccagcgttccttctctaca
 L  S  S  S  V  P  S  L
```

B

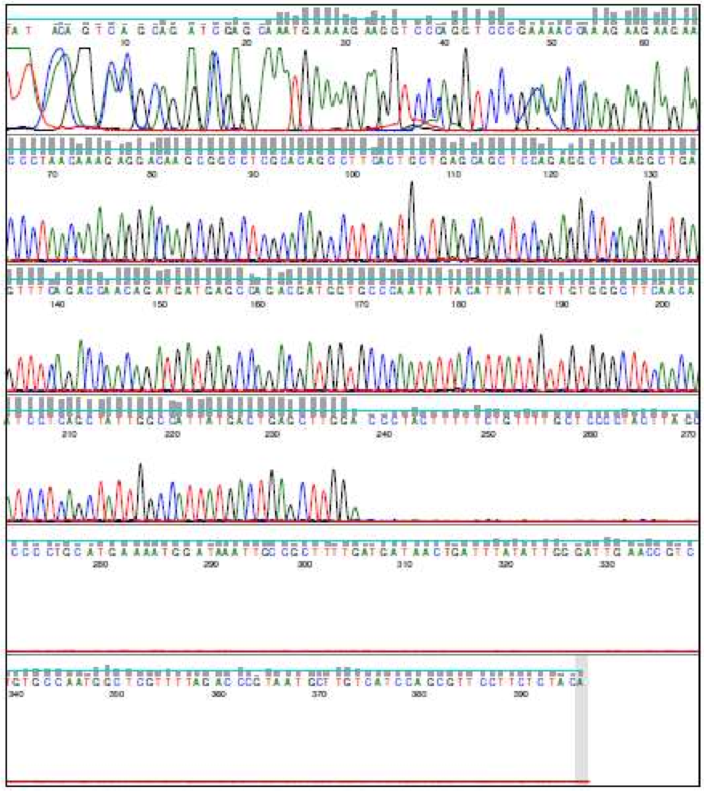

Supplement: Supplementary file 1 [file ijms-19-01252-s001.zip › ijms-287252 supplementary/Supplementary figure 2.pdf]
