# Supplementary material for: SPAG17 Is Required for Male Germ Cell Differentiation and Fertility
Source: Int J Mol Sci. 2018 Apr 21;19(4):1252. doi: 10.3390/ijms19041252 (PMC5979577; doi:10.3390/ijms19041252)
Supplement: Supplementary file 1 [file ijms-19-01252-s001.zip › ijms-287252 supplementary/Supplementary table 1.docx]

Supplementary table 1: List of antibodies used for this study.

| Antibody | Reference | Dilution | Sequence |
| --- | --- | --- | --- |
| Anti-SPAG17 4915 | Zhang et al., 2005 | 1/200 | EQEMQSKLPLWEFLQFPLPPPWNSTKRLATIHELMHFCTNEVLSWNEVERAFKVFTFESLKLSEVDEEGRLKPTETTSDTDVENFNIPWDNPARFAKLIRQRYIHRMSMQKAPPVVVEIENTERTLFVNKNFAKAEQDAQGDENSPNSDEPDAISVTGSTSNSTKPWNSSNRQFSEKETSGSMWPQPESMDQTMDTEIKDDAATKDDSPEKKPKKMVVEADIEDIKKTQQRSLMDWSFTEYFQPKVLLQVLQEAHQQYRCVDSYYHTQDNSLLLVFHNPMNLQRLQCEHWNIALHSNVGFRNYLELVAKSIEDWVTQEEAKYQEAKMAEELNRIRIELELKAT |
| Anti-Pcdp1 | Lee et al.,2008;  Teves et al., 2013 | 1/100 | KNLRSKALNTYLILD |
| Anti-Acetylated Tubulin | Sigma-Aldrich  #T6793 | 1/200 | N/A |
| Anti-IFT20 | Proteintech#13615-AP | 1/50 | N/A |
