# Supplementary figures and images for: SPAG17 Is Required for Male Germ Cell Differentiation and Fertility
Source: Int J Mol Sci. 2018 Apr 21;19(4):1252. doi: 10.3390/ijms19041252 (PMC5979577; doi:10.3390/ijms19041252)

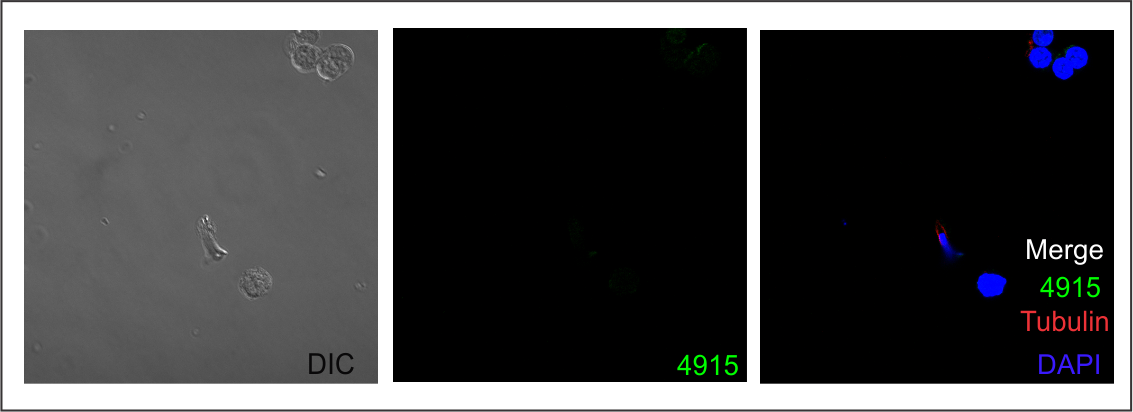

Supplement: Supplementary file 1 [file ijms-19-01252-s001.zip › ijms-287252 supplementary/Supplementary figure 1.tif]
